# Innate immunity against HIV: a priority target for HIV prevention research

**DOI:** 10.1186/1742-4690-7-84

**Published:** 2010-10-11

**Authors:** Persephone Borrow, Robin J Shattock, Annapurna Vyakarnam

**Affiliations:** 1 Nuffield Department of Clinical Medicine, University of Oxford, The Jenner Institute, Compton, Newbury, Berkshire RG20 7NN, UK; 2 Department of Cellular and Molecular Medicine, St George's, University of London, Tooting, London, SW17 ORE, UK; 3 Department of Infectious Diseases, King's College London, 2nd Floor, New Guy's House, Guy's Campus, London SE1 9NU, UK

## Abstract

This review summarizes recent advances and current gaps in understanding of innate immunity to human immunodeficiency virus (HIV) infection, and identifies key scientific priorities to enable application of this knowledge to the development of novel prevention strategies (vaccines and microbicides). It builds on productive discussion and new data arising out of a workshop on innate immunity against HIV held at the European Commission in Brussels, together with recent observations from the literature.

Increasing evidence suggests that innate responses are key determinants of the outcome of HIV infection, influencing critical events in the earliest stages of infection including the efficiency of mucosal HIV transmission, establishment of initial foci of infection and local virus replication/spread as well as virus dissemination, the ensuing acute burst of viral replication, and the persisting viral load established. They also impact on the subsequent level of ongoing viral replication and rate of disease progression. Modulation of innate immunity thus has the potential to constitute a powerful effector strategy to complement traditional approaches to HIV prophylaxis and therapy. Importantly, there is increasing evidence to suggest that many arms of the innate response play both protective and pathogenic roles in HIV infection. Consequently, understanding the contributions made by components of the host innate response to HIV acquisition/spread versus control is a critical pre-requisite for the employment of innate immunity in vaccine or microbicide design, so that appropriate responses can be targeted for up- or down-modulation. There is also an important need to understand the mechanisms via which innate responses are triggered and mediate their activity, and to define the structure-function relationships of individual innate factors, so that they can be selectively exploited or inhibited. Finally, strategies for achieving modulation of innate functions need to be developed and subjected to rigorous testing to ensure that they achieve the desired level of protection without stimulation of immunopathological effects. Priority areas are identified where there are opportunities to accelerate the translation of recent gains in understanding of innate immunity into the design of improved or novel vaccine and microbicide strategies against HIV infection.

## Understanding how innate immunity modifies HIV infection offers unique opportunities for the development of novel prophylactic and therapeutic strategies

Rational approaches to HIV vaccine design have so far focused principally on the induction of virus-specific antibody or T cell responses. Results from large-scale clinical trials of both antibody- and T cell-targeted immunogens have given largely disappointing results [1,2] and although some short-lived protection was observed in the most recent phase III HIV vaccine trial [3], the mechanism(s) of protection are not well understood. There is thus an urgent need for novel approaches to HIV prophylaxis and therapy that will complement and synergise with traditional strategies centred on stimulation of adaptive responses.

The classical application of innate immunity in vaccine design has been in an adjuvant role: innate immune responses are stimulated at the time of vaccination to promote the induction of adaptive response(s) capable of mediating protection on subsequent pathogen encounter [4]. The need for a better understanding of links between innate and adaptive immunity and of the type(s) of innate response that should be stimulated to prime protective responses, particularly at mucosal sites, are discussed in a separate report [5]. However a second, more novel means of applying innate immunity in prevention strategies (vaccine and microbicides) would be in an effector capacity: i.e. to stimulate innate or adaptive responses that would modulate the innate responses activated at the time of subsequent pathogen exposure to provide (or contribute to) protection. This review focuses on opportunities for applying the latter type of strategy in the development of novel approaches to prevention of HIV infection.

Understanding the contributions made by different innate host resistance mechanisms and innate responses to HIV acquisition and disease progression is a critical pre-requisite for the rational design of novel prophylactic and therapeutic strategies focusing on innate immunity: this will inform the selection of responses to target for up- or down-modulation by vaccination or microbicides. There is also an important need to understand the mechanisms via which innate responses are triggered, so that these can be selectively exploited or inhibited in vaccine or microbicide design. Finally, strategies for achieving the desired modulation of innate functions will need to be developed and subjected to rigorous testing to ensure that they achieve the desired level of protection without stimulation of immunopathological effects. Given that many components of the innate response mediate pleiotropic functions and can both inhibit HIV infection and exert immunomodulatory effects that may enhance viral replication, it is critical to assess whether these opposing outcomes can be dissected and mapped to functionally distinct effector pathways or sites within a given soluble factor, thereby providing a basis for their selective exploitation in prophylactic or therapeutic strategies.

## Innate responses in HIV infection and their roles in protection or pathogenesis

**Figure 1:**
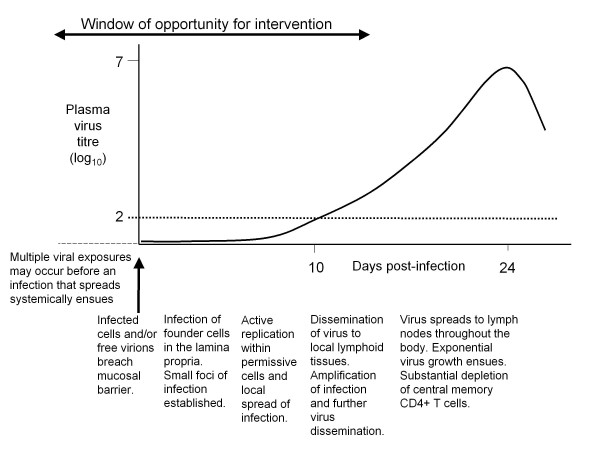
**Sequence of events during the eclipse and viral expansion phases of acute HIV-1 infection**. Mucosal transmission of HIV is followed by an eclipse phase of ~ 10 days during which small foci of infection are established in the mucosa, local virus replication occurs and infection spreads to local lymphoid tissues where further virus amplification takes place. More widespread virus dissemination then ensues, with infection of lymph nodes throughout the body including the GALT where high levels of virus replication take place, associated with an exponential increase in plasma viral titres. The horizontal dotted line indicates the limit of detection of many of the assays conventionally used to evaluate plasma HIV titres (~100 viral RNA copies/ml): the time at which this is exceeded constitutes the end of the eclipse phase. As illustrated, there is a relatively short window of opportunity during which infection could potentially be blocked, eradicated or constrained before substantial CD4+ T cell depletion occurs and the stage is set for subsequent disease progression.

The following sections discuss current understanding of the roles of different components of innate immunity in protection or pathogenesis in HIV infection and of how the activation of innate responses is stimulated and regulated, together with the knowledge gaps and priorities for research. Components of the innate response are considered in the sequence in which they may be invoked in combating infection: as mucosal HIV exposure occurs; local foci of infection are established; and as more widespread viral dissemination takes place (Figure 1).

### a. The importance of innate defences in forming barriers to or conversely promoting mucosal HIV infection

The observation that following heterosexual transmission of HIV, the viral quasispecies generated in acute infection is frequently derived from a single infecting virion [6], provides support for the existence of robust barriers to HIV infection via the genital mucosa. These barriers are in part physical (mucus, low pH, epithelial integrity), but in addition there are a number of secreted factors present at the genital mucosa that display anti-HIV activity (or possess infection-enhancing properties), many of which can in turn be modulated by HIV infection. Broadly, these factors fall into two groups: (i) cationic peptides and (ii) small secreted proteins. In addition to having a direct impact on HIV infectivity, many of these factors also mediate innate immunomodulatory activity and consequently have the potential to impact innate and adaptive immune responses more broadly. Examples include a peptide in semen named semen-derived enhancer of virus infection (SEVI), defensins, members of the cysteine-rich whey acidic protein (WAP) family, and type I interferons (IFNs).

The molecular mode of action of many of these recently-discovered factors remains to be elucidated, with indications from published data highlighting substantial diversity in potency and mode of interaction with HIV. For example, SEVI, a small semen cationic peptide, enhances HIV infection *in vitro *under conditions designed to mimic those encountered during sexual transmission of HIV through formation of amyloid fibrils that capture and focus virus onto target cells [7-9]. Understanding precisely how this peptide self-aggregates to form β-sheet-rich amyloid fibrils and how this process may be disrupted could improve the potential to reduce HIV transmission.

Defensins are also small cationic peptides. They are produced by epithelial cells and leukocytes and are involved in combating infection with a broad range of bacteria, fungi and viruses, including HIV [10]. Mechanisms proposed to contribute to their anti-HIV activity include direct inactivation of virions, interference with attachment/entry via impairment of gp120 binding to CD4, co-receptor down-regulation, induction of β-chemokines or inhibition of the fusion step and down-regulation of viral replication at an intracellular level [11-16]. Certain α-defensins may also enhance HIV infection by promoting viral entry through an unknown mechanism [17]. Notably, defensins also mediate immunomodulatory effects, acting as chemoattractants for T cells, monocytes and dendritic cells (DCs) and regulating cellular activation and cytokine production [18-22]. These immune-stimulatory properties of defensins could help to enhance acquisition of HIV infection by increasing the availability of infection-susceptible target cells at mucosal exposure sites. Whether the pro- or anti-HIV activities of defensins predominate *in vivo *is not clear, although local elevations in α-defensin levels during genital tract infections are associated with enhanced HIV acquisition [17,23]. Analysis of the propensity of different defensins to mediate these diverse activities and dissection of structure-function relationships could potentially enable the development of microbicides that selectively employ the HIV-inhibitory properties of defensins to reduce mucosal HIV transmission.

Whey acidic proteins have traditionally been associated with broad antimicrobial activity at portals of pathogen entry and are identified to be under strong selection pressure, which is a hallmark of innate immunity [24]. Two of the 18 human family members, secretory leukocyte protease inhibitor (SLPI) and Elafin display anti-HIV activity, correlating with reduced virus transmission [25-28]; however, a third member, whey acidic protein four-disulfide core domain 1 (WFDC1)/ps20, expressed in several mucosal tissues, enhances HIV infection [29]. SLPI exerts an anti-HIV effect by binding to annexin II (a cell surface cofactor that binds phosphatidylserine and promotes HIV entry by stabilising virus fusion beyond the HIV receptor/co-receptor complex) and impairing annexin II-mediated stabilisation of fusion [27,28]. The mechanism underlying the antiviral effect of Elafin, which is over-expressed in female genital tract of highly exposed uninfected individuals, is unknown [25]. WFDC1/ps20 promotes infection by a method that appears in keeping with a more fundamental biologic role of this factor in promoting cell adhesion and regulation of the extracellular matrix. Ps20-upregulation of CD54 expression and possibly other adhesion antigens and tetraspanins involved in the formation of the virological synapse [30] is postulated to promote cell-free and cell-cell virus transfer ([30] and Vyakarnam *et al*., submitted). How ps20 regulates cell adhesion and HIV infection is not known. In addition to their ability to regulate HIV infection, whey acidic proteins are recognised for their anti-inflammatory activity, e.g. they are able to suppress lipopolysaccharide (LPS)-stimulated production of cytokines like tumour necrosis factor (TNF)α [30]; and ps20 can suppress toll-like receptor (TLR)3-mediated induction of IFNα in human cells, which may contribute to its infection-enhancing activity (Vyakarnam *et al*., unpublished). SLPI has also been noted to suppress the enzyme activation-induced cytidine deaminase (AID) in epithelial cells [31]. AID is important for B cell receptor editing and immunoglobulin (Ig) class switching [32]. Epithelial cells express both AID and SLPI upon sensing pathogen products through TLRs [31]. SLPI in turn attenuates AID activity via nuclear factor (NF)-κB down-modulation [31]. The molecular mechanisms that underpin these functions of whey acidic proteins are not known, but are of priority to understand, particularly given the importance of local immune activation in enhancing acquisition of HIV infection and the subsequent importance of systemic immune activation in promoting HIV replication both during early and in established infection (where damage to the gut-associated lymphoid tissue (GALT) leading to enhanced bacterial translocation and increased circulating LPS levels has been proposed to be a significant cause of ongoing immune activation [33,34]). Maintaining circulating levels of whey acidic proteins may therefore be important in limiting immune activation throughout infection [35].

Type I IFNs are innate cytokines that also possess direct anti-HIV activity [36] and, like many of the factors discussed above, have multiple other effects, including regulation of immune activation and cellular apoptosis. They mediate their pleiotropic activities by binding to a common receptor and triggering different intracellular signalling cascades that result in transcriptional up-regulation of IFN-stimulated genes. The host cell functions regulated by type 1 IFNs include an array of antiviral mechanisms that act to block HIV replication at multiple stages in the viral life-cycle [37]. Type 1 IFNs in mucosal secretions may thus help to maintain a "baseline" level of HIV resistance in cells at local sites of viral exposure. However these innate cytokines also possess potent immunostimulatory properties, promoting the activation and functional maturation of multiple cell types including DCs, macrophages, natural killer (NK) cells and T cells [38]. As local immune activation can enhance HIV infection, the presence of type 1 IFNs at mucosal sites could also have detrimental consequences. Which prevail *in vivo *is currently unclear. Likewise type 1 IFN induction following HIV transmission as infection is established and begins to spread, and its production at subsequent stages of infection could also have opposing effects - this is discussed further below.

**Figure 2:**
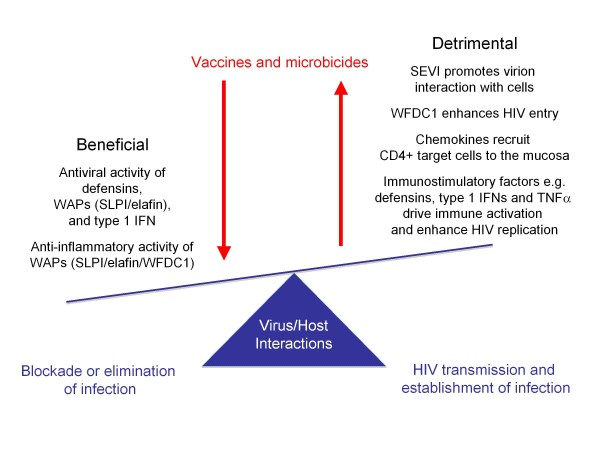
**Opposing effects of soluble factors present at mucosal sites of HIV exposure on virus transmission and the establishment of infection**. As illustrated, soluble factors at mucosal sites can mediate beneficial effects by exerting direct antiviral activity or reducing local inflammation; and/or can mediate detrimental effects by enhancing virus transmission, directly augmenting HIV infection of cells, recruiting CD4+ target cells or promoting local immune activation/increasing HIV replication. Vaccines and microbicides should be designed to tip the balance in favour of the beneficial effects.

Taken together these data highlight that innate immune mediators present at local sites of infection can exert a significant HIV regulatory effect through physical interaction with the virus, competitive binding to cell-surface entry proteins, triggering of signals that alter the permissiveness of target cells and/or immunomodulatory activities (Figure 2). At present there are significant gaps in our understanding of the molecular mechanisms underlying these effects. Systematic study of the structure/function relationship of these factors, delineation of their mode of action (where appropriate through identifying binding/signalling partners that link immunoregulatory function to HIV regulatory activity) and determination of how HIV regulates the expression of these proteins in *in vitro *model systems are critical. In addition, development of specific assays for the accurate measurement of these secreted innate factors will enable their regulation and expression pattern in mucosal tissue during acute and chronic infection to be assessed. Together, this will provide a platform for considering the potential exploitation of these innate immune mediators in novel prophylactic or therapeutic strategies.

### b. Cellular HIV restriction factors and their modulation in primary cells

Human cells express a number of proteins that block cross-species transmission of retroviruses [39]. Some of these species restriction factors, namely apolipoprotein B editing complex, catalytic subunit (APOBEC)3G/F [40], tripartite motif (TRIM)5α [41] and tetherin [42], display broad antiviral effects in over-expression model systems. Virus-host adaptation has led to HIV evolving specific mechanisms to counteract the action of these potent species restriction factors. Indeed replication-competent strains of HIV carry specific virally-encoded accessory genes that have evolved to counteract APOBEC3G and tetherin anti-HIV activity [43,44], thereby ensuring their propagation in human cells. A significant body of research is currently focused on understanding the molecular mechanisms by which HIV interacts directly with these restriction factors and overcomes their antiviral effects, which has potential implications for the development of novel antivirals. This area of research is outside the scope of this review.

HIV restriction factors are constitutively expressed at baseline levels in many cell types, but their expression can be rapidly up-regulated by type 1 IFNs [45-49]. Up-regulation of these restriction factors may account for much of the anti-HIV-1 activity of type 1 IFNs, although there is evidence to suggest that "classical" IFN-induced antiviral pathways may also contribute to control of HIV replication [44]. Type 1 IFNs inhibit HIV replication in both CD4+ T cells and macrophages, but their ability to block viral replication in the latter cells is much more profound [50]. In line with this, it is notable that HIV infection of macrophages fails to induce type 1 IFN production [51] (thought to be due to lack of high-level expression of TLR7 or other pattern recognition receptors capable of recognising HIV and triggering type 1 IFN production [52]); and that HIV infection of CD4+ T cells is associated with depletion of interferon-regulatory factor-3 (IRF-3), which impairs IFN induction through the retinoic acid-inducible gene I (RIG-I) pathway [53]. The fact that HIV hardly triggers type 1 IFN production in infected cells could be a reflection of its need to avoid the potent antiviral activity of IFN-induced HIV restriction factors.

A key question, yet to be answered, is whether increased expression of endogenous restriction factors could prevent HIV infection or limit virus replication in acute/early infection. Interestingly, a recent study suggested that APOBEC3G expression can be modified by vaccination. Rectal mucosal immunization of macaques with SIV antigens and CCR5 peptides, linked to the 70 kD heat shock protein, showed a progressive increase in APOBEC3G mRNA in PBMCs which was maintained for at least 17 weeks. Mucosal challenge with simian immunodeficiency virus (SIV) resulted in a significant increase in APOBEC3G mRNA in CD4+CCR5+ cells in the circulation and draining iliac lymph nodes in immunized animals (which did not become infected) compared to un-immunised animals, consistent with an association between APOBEC3G expression and protection from infection [54]. However it remains unclear whether the increase in APOBEC3G expression limited HIV infection *per se*, or provided a surrogate marker for IFN induction, which was mediating its effects via a variety of mechanisms. This question is of importance in the context of understanding and exploiting innate immune effector mechanisms in therapeutic strategies. A recent study in a murine model system showed that type 1 IFN-mediated suppression of the replication of hepatitis B virus (which is also sensitive to the antiviral effects of APOBEC3) was not dependent on APOBEC3 expression [55]. A systematic analysis of the regulation of restriction factors by innate immune mediators like IFNs and whey acidic proteins, combined with functional studies of HIV infection in human cells treated with innate immune mediators in which restriction factors have been silenced through siRNA-mediated knockdown techniques would provide a better understanding of the role that HIV restriction factors play a role in innate immunity to HIV-1 infection.

### c. The role of dendritic cells and macrophages at local (mucosal) sites in promoting or restricting establishment of initial foci of infection and virus dissemination

The low efficiency of heterosexual HIV transmission may be due not only to the physical and immunological barriers to infection at genital mucosal surfaces (discussed above), but also to difficulties encountered by the virus in establishing initial foci of infection at the local mucosal site and undergoing subsequent spread. A full understanding of the earliest virus-host cell interactions that take place in infection and the role of local innate responses in blocking or amplifying initial virus replication is paramount to enable the development of prophylactic strategies to intervene at this critical stage of infection where the window of opportunity for virus eradication is still open.

The first cells with which the virus interacts at the genital mucosa may include DCs, macrophages and CD4+ T cells. Conventional (c)DCs in the submucosa expressing the C-type lectin dendritic cell-specific, intercellular adhesion molecule-grabbing non-integrin (DC-SIGN) are hypothesized to play a key role in HIV dissemination to CD4+ T cells due to their ability to capture and internalize virions via DC-SIGN and mediate trans-infection of CD4+ T cells, either at the mucosal infection site or following migration into draining lymphoid tissues. Some macrophages in mucosal tissues also express DC-SIGN and, although they do not migrate into lymph nodes, may contribute to local HIV transmission to CD4+ T cells [56]. HIV interaction with DC-SIGN also has a number of other important consequences. It stimulates leukemia associated Rho guanine nucleotide-exchange factor (LARG)-induced Rho-GTPase activation, which promotes virus-T cell synapse formation and increases virus replication [57]. It also leads to activation of Raf-1, which together with TLR8-stimulated NF-κB activation is required to enable HIV to replicate in DCs [58]. LARG-induced Rho-GTPase activation and Raf-1 activation both also have immunomodulatory effects, the former causing down-regulation of major histocompatibility complex (MHC) class II molecules and IFN response genes in monocyte-derived DCs [57] and the latter modulating the cytokine response induced following TLR ligation, notably increasing production of pro-inflammatory cytokines [59]. By binding to pattern-recognition receptors (PRRs) including DC-SIGN, HIV-1 may thus simultaneously achieve amplification, dissemination and subversion of the host immune response to further its replication and spread.

Langerhans cells located in the mucosal epithelium express an alternative capture receptor, Langerin. In contrast to virions captured by DC-SIGN, HIV-1 captured by Langerin is internalised into Birbeck granules and degraded [60]. Unlike DC-SIGN+ cDCs in the subepithelium, Langerhans cells may thus bring about clearance of captured virions rather than mediating HIV transmission to T cells. However, if Langerhans cells are activated, e.g. as a consequence of local infection with other pathogens, they mediate transinfection rather than virion destruction [61]. There is also evidence for the existence of additional HIV capture receptors, whose functions are less well understood [62]. Better characterisation of the full array of receptors expressed by Langerhans cells and submucosal DCs (as well as other cDC and macrophage subsets) and their roles in mediating virion destruction, transinfection, and intracellular signaling to modulate DC functions is an area of importance for future work.

Acquisition of HIV infection is known to be enhanced by the presence of other sexually transmitted infections. Langerhans cell activation may be only one of a number of mechanisms involved, others including breach of the physical mucosal barrier to infection, the presence of larger-than-normal numbers of CD4+ T cells, macrophages and DCs in the subepithelium, and the heightened state of activation of these cells. In the resting state, the paucity of CD4+ T cells in the submucosa may be one of the factors that restricts the establishment of foci of HIV infection. Recent studies in the SIV macaque model have suggested that production of macrophage inflammatory protein (MIP)-3α at mucosal infection sites may attract plasmacytoid (p)DCs and other inflammatory cells, which in turn help to recruit additional CD4+ T cells through production of chemokines such as MIP1α and β [63]. These pathways are potential targets for intervention strategies, and require further investigation. As potent sources of type 1 IFN production, pDCs also have the capacity to combat HIV replication at local infection sites. The opposing roles of pDCs in restricting and potentiating initial virus replication and the factors that govern which of these activities predominate, including the role of HIV signaling through TLRs and other pattern recognition receptors and how this affects pDC functions, are also of importance to understand.

### d. HIV-DC interactions as virus dissemination starts to occur: impact on systemic activation of innate and adaptive responses

DCs play a key role in the orchestration of innate and adaptive responses, responding to the presence of infection by initiating soluble factor production and engaging in cross-talk with other cell types to induce and regulate an immune response that will mediate pathogen control with minimum immunopathology. Given the central role of DCs in the host immune response, many viruses subvert DC functions to promote their persistence *in vivo*. HIV is no exception: it exploits DC subsets not only to facilitate the initial establishment of infection and local virus spread as described above, but also to enhance systemic virus replication and impair host control of infection.

HIV can infect both cDCs and pDCs, and has been found to initiate infection in both DC subsets more efficiently than in other cell types (including macrophages and CD4+ T cells) *in vitro *[64]. However the frequency of infected DCs that produce virus is low, likely due to the fact that DCs express HIV restriction factors, e.g. monocyte-derived DCs express APOBEC3G/3F, levels of which are up-regulated as they mature [65]. Nonetheless, although DCs probably do not consitute a major cellular site for HIV production, they promote systemic HIV replication in two important ways. First, they mediate transfer of infection to CD4+ T cells, particularly to the antigen-specific CD4+ T cells with which they interact [66], thus simultaneously driving virus amplification and impairment of the HIV-specific CD4+ T cell response. cDCs may be particularly important in this regard, as although pDCs transfer HIV to CD4+ T cells too they also produce type 1 IFNs that block virus replication in T cells [67]. Second, DCs activated by HIV stimulate a high level of generalised immune activation that provides the activated target cells required for optimal disseminated HIV replication. The importance of this is increasingly appreciated.

*In vitro *studies have shown that pDCs are very rapidly activated following contact with HIV, upregulating MHC and costimulatory molecules, producing high levels of type 1 IFNs and other cytokines and acquiring increased T cell stimulatory capacity [68]. HIV stimulates pDC activation by a process that involves virion endocytosis following binding to CD4 and CCR5 and subsequent ligation of TLR7 by HIV RNA [68]. By contrast, cDCs do not undergo a similar functional activation on exposure to HIV, despite the fact that they can bind HIV via CD4 and CCR5 and also express TLR7 and are activated by TLR7 agonists including HIV RNA sequences [69]. The reasons for this are not fully understood, but HIV may be routed into different intracellular compartments in cDCs and/or cDC activation may be blocked by signals delivered through PRRs other than TLR7. Although cDCs are not directly activated by HIV, they nonetheless undergo bystander maturation in the presence of HIV-exposed pDCs [70]. These *in vitro *findings would predict a rapid widespread activation of pDCs and subsequent maturation of cDCs as systemic HIV spread occurs: a picture which fits well with observations made *in vivo*.

**Figure 3:**
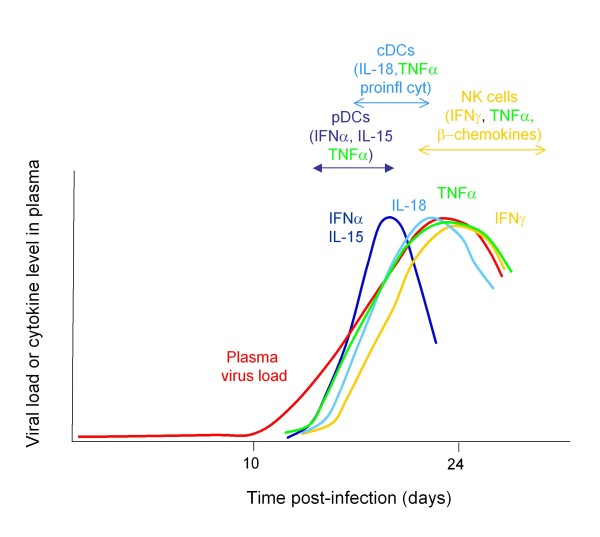
**Diagram to illustrate the kinetics of activation of systemic innate responses during acute HIV-1 infection**. The exponential increase in plasma viral titres (red line) is associated with elevations in circulating levels of a multiple cytokines and chemokines (coloured lines), which likely reflect the systemic activation of pDCs, cDCs, macrophages, NK cells and other cell types.

The increase in plasma viral titres in acute HIV-1 infection (AHI) is associated with an ordered sequence of elevations in systemic levels of multiple cytokines and chemokines [71]. The earliest systemic cytokine elevations include rapid and transient increases in plasma levels of IFNα and interleukin (IL)-15, a rapid but more sustained increase in TNFα, and a slightly slower but also more sustained increase in IL-18 accompanied by elevations in multiple other pro-inflammatory cytokines/chemokines, and a late-peaking increase in IL-10 [71]. The initial pattern of cytokine elevations would be consistent with systemic activation of pDCs to produce IFNα, IL-15 and TNFα as viremic HIV spread occurs, followed by initiation of TNFα and IL-18 production by cDCs and induction of further pro-inflammatory cytokine/chemokine production by other cell types (Figure 3). The cellular sources of acute-phase cytokine production and the role of pDCs in initiating the acute-phase cytokine storm remain to be confirmed; but rapid activation of circulating DCs as viremic HIV spread takes place is also suggested by the marked reduction in cirulating pDC and cDC numbers that occurs prior to the peak in HIV viremia [72]. Studies in rhesus macaques acutely infected with SIV suggest that this is due to migration of activated pDCs to lymph nodes [73] where they undergo death as a consequence of activation, infection and/or exposure to pro-apoptotic signals [74]. Accumulation of cDCs with a partly-activated phenotype in lymphoid tissues has also been observed in AHI [75]. It is notable that the increase in plasma viral titres in the acute phase of hepatitis B and C virus infections is not accompanied by high-magnitude elevations in circulating type 1 IFN levels and induction of a systemic cytokine storm equivalent to that observed in AHI [71]. HCV does not stimulate pDCs to produce high levels of type 1 IFNs *in vitro*, and impairs their response to TLR7 and TLR9 ligation [76,77]. This is in marked contrast to the pronounced pDC-stimulatory capacity of HIV, and would support a key role for the latter in induction of the florid systemic cytokine response observed in AHI.

Given the ability of type 1 IFNs to inhibit HIV replication *in vitro *(discussed above), it seems likely that these antiviral cytokines also contribute to control of HIV replication *in vivo*, although the extent to which they constrain the acute viral burst and the mechanisms by which they mediate this are important issues which require investigation. In addition to their antiviral effects type 1 IFNs and other innate cytokines such as IL-15 also possess potent immunostimulatory properties, mediating immune activation both directly and indirectly (via induction of other cyokines/chemokines) - hence they may contribute to control of viral replication indirectly, by activating other protective immune responses. However they may simultaneously act to enhance HIV replication by driving widespread immune activation; further, type 1 IFNs promote apoptosis, so they may also contribute to CD4+ T cell loss in HIV infection [78]. Which of these activities predominates *in vivo *remains unclear - although the fact that HIV has not evolved to avoid or inhibit pDC activation suggests that on balance the early innate cytokine production and ensuing immune activation may be advantageous for virus replication. In support of this, administration of IL-15 to rhesus macaques during the acute phase of SIV infection resulted in enhancement of NK cell and SIV-specific CD8+ T cell responses at peak viremia and a reduction in the number of SIV-infected cells in lymph nodes, but despite this, establishment of higher persisting viral loads and enhanced disease progression [79]. A recent study showed that pDCs from women produce more IFNα following stimulation with TLR7 ligands derived from HIV RNA than pDCs from men [80]. In early HIV-1 infection, viral loads in women tend to be lower than those in men, but in chronically-infected subjects with a given viral load women tend to progress to AIDS faster than men. It is thus possible that cytokine production by pDCs may have beneficial effects in the early stages of infection, but subsequently promote disease progression - although other sex-related effects may also contribute to these differences in HIV control [80].

Analysis of the extent and kinetics of type 1 IFN induction and immune activation stimulated during SIV infections of their natural non-human primate hosts, which unlike HIV infection in humans generally do not lead to the development of AIDS, has also helped to give insight into what may consititute more protective versus pathogenic aspects of the early immune response. Most studies suggest that pDC activation, type 1 IFN production and immune activation occur in the acute phase of both pathogenic and non-pathogenic SIV infections, but non-pathogenic infections are distinguished by the fact that the acute-phase immune activation resolves very rapidly even though substantial virus replication continues after the transition to chronic infection [81-85]. By contrast in HIV infection and pathogenic SIV infections a chronic state of immune activation is established, the level of which is predictive of the subsequent rate of disease progression [86-88]. Notably, sustained TLR7 triggering in mice has been shown to induce chronic immune activation and progressive lymphoid system disruption with similarities to that in HIV infection [89]. In addition to the difference in the duration of immune activation in pathogenic and non-pathogenic infections, some studies also suggest that the extent of pDC activation and level of type 1 IFN production in the acute phase of infection may be related to subsequent disease progression [90,91]. Identification of the mechanisms responsible for the more limited and/or more rapidly down-regulated immune activation in nonpathogenic SIV infections (which may comprise host immunomodulatory mechanisms [92] and/or effects of specific viral proteins [93]) is an important priority for future studies.

Although HIV stimulates pDC activation, hence promoting a state of generalised immune activation that permits widespread high-level virus replication, increasing evidence suggests that it also modifies both pDC and cDC functions in order to concurrently reduce or impair the activation of virus-specific T cell responses. Although cDCs are not directly activated by HIV, exposure to HIV impairs their maturation in response to other stimuli and promotes production of IL-10 and induction of a regulatory T (Treg) cell response [94]. In addition, when HIV activates pDCs, it stimulates production of indoleamine 2,3-dioxygenase (IDO) [95]. HIV-stimulated pDCs induce the differentiation of naïve CD4+ T cells into Treg cells with suppressive functions via an IDO-dependent mechanism [96]. IDO activity has been shown to be upregulated concurrently with IFNα production and pDC accumulation in lymph nodes during acute SIV infection in macaques, and to be negatively correlated with SIV-specific CD4+ T cell proliferation [73]. However the extent to which this occurs during the acute phase of HIV infection and its impact on the HIV-specific T cell response remain to be determined. It is also important to dissect the pathways by which HIV mediates these alterations in DC functions, so that strategies can be designed to block them. Another important question is to what extent *in vivo *impairments in DC-T cell interactions can be overcome by pre-priming of HIV-specific T cell responses.

### e. NK and NKT cell activation and functions in HIV infection

NK and NKT cells are innate lymphocyte populations that can be rapidly activated in response to infection and are capable of mediating potent effector and immunoregulatory functions. As such, they warrant consideration in HIV vaccine design, where modulation of events taking place in the earliest stages of infection is paramount. A better understanding of the NK and NKT cell responses activated following HIV infection and their contributions to control of viral replication and/or to immunopathological immune activation is thus a current priority.

#### NK cells

In AHI, peripheral blood NK cells become activated and increase in frequency as the acute burst of viral replication occurs, prior to the maximal expansion of CD8+ T cells [97]. NK cells also exhibit enhanced activity *ex vivo *(degranulation and cytokine production) at this time, a property that is sustained throughout early infection. Interesting changes take place in the peripheral blood NK cell subset composition during AHI. The frequency of CD56bright (regulatory) NK cells decreases (perhaps due in part to recruitment into lymph nodes), whilst the frequency of CD56dim (effector) NK cells increases [97]. Notably, there is also a selective increase within the effector NK population in the frequency of cells expressing the activating receptor KIR3DS1 in individuals who co-express HLA class I molecules with the HLA-Bw480I motif, the putative class I ligand for KIR3DL1/S1 [98]. The mechanisms involved in selective KIR3DS1 + NK cell expansion/survival in acute and early infection remain to be determined, but are of importance from a vaccine design perspective.

There is increasing evidence to suggest that NK cells make a significant contribution to containment of viral replication in HIV-infected individuals. NK cells are able to control HIV replication *in vitro*; and the observation that HIV has evolved strategies for modulating ligand expression on the surface of the cells it infects so as to minimize both CD8+ T cell and NK cell activation but maximise NK cell inhibition suggests that NK cells also mediate antiviral activity *in vivo *[99,100]. Notably, KIR3DS1+ NK cells are particularly potent inhibitors of HIV replication in HLA-Bw480I-positive target cells *in vitro *[101]. Genetic studies also provide support for a role for KIR3DS1+ and KIR3DL1+ NK cells in control of HIV replication *in vivo*: co-expression of *HLA-Bw480I *with *KIR3DS1 *or certain inhibitory alleles of *KIR3DL1 *has been found to be associated with low-level viremia in early HIV infection and also with delayed disease progression [102-104]. Likewise associations have been reported between *KIR3DL *alleles and viral loads in SIV-infected rhesus macaques [105]. NK cells may also help to mediate resistance to HIV infection, as enhanced NK cell activity has been reported in HIV-exposed, seronegative individuals [106,107], an observation suggested to be due to the balance of activating/inhibitory receptor expression on their NK cells [108,109]. Although these studies provide an initial indication that NK cells possess anti-HIV effector activity that may have potential for exploitation in HIV vaccine design, there are many important questions that remain to be answered.

First, why do some NK cells, e.g. those expressing KIR3DS1 or certain KIR3DL1 allotypes, seem to be particularly protective in HIV infection? If the reasons for this were understood, it may be possible to design strategies to induce NK cells in people who do not express these receptors or their ligands to mediate better control of HIV replication (e.g. by modulating signalling through other receptors to mimic the effects of the protective KIRs). It is hypothesized that the activating receptor KIR3DS1 interacts with a specific ligand on HIV-infected cells that results in efficient triggering of effector functions. However the nature of this ligand and reasons for the efficacy of NK stimulation via KIR3DS1 remain unclear. Whether HIV-infected cells can express ligands for other activating KIRs also remains to be determined. KIR3DL1 is an inhibitory receptor, and is hypothesized to act during NK cell development/functional maturation to permit NK cells to acquire particularly powerful effector functions. In line with this, KIR3DL1+ NK cells from *HLA-Bw480I *subjects respond strongly to stimulation with HLA-deficient cells *in vitro *[110]. However the processes involved in NK cell development/maturation are not fully understood, and the action of KIR3DL1 and possible approaches for mimicking it require further investigation.

Second, although recent studies have begun to give insight into the systemic activation of NK cells in HIV infection, relatively little is known about the NK cell populations present at other sites, such as the genital mucosa, the gut and lymph nodes, and the roles they may be playing in combating local HIV replication and/or mediating immunopathological effects. Of particular interest is the recently-described IL-22-producing NK population in the gut, which may play an important role in mucosal defence and local immunoregulation [111].

Third, it is critical to understand whether NK cells have immunopathological effects in addition to their putative protective functions in acute and early HIV infection. NK cells may contribute to immunopathological CD4+ T cell destruction, e.g. a role for NKp44-expressing NK cells in mediating lysis of uninfected CD4+ T cells expressing a gp41 peptide-induced NKp44 ligand has been suggested [112]. NK cells may also promote immune activation via either direct or indirect mechanisms, hence enhancing viral replication and spread. The observation that individuals with KIRs encoded on the B group of KIR haplotypes (which contain multiple activating KIRs) undergo more rapid disease progression in chronic HIV infection than subjects who only have KIRs encoded on the A group of KIR haplotypes [113] would be consistent with a link between higher levels of NK activation and promotion of viral replication. Further, in early HIV infection, higher levels of NK cell functional activity are observed in *KIR3DS1+ *compared to *KIR3DS1- *individuals, independent of expression of *HLA-Bw480I *[114]; and although subjects who express both *KIR3DS1 *and *HLA-Bw480I *progress to AIDS slowly, *KIR3DS1 *homozygosity in the absence of *HLA-Bw480I *expression is associated with accelerated disease progression [104]. This could mean that if the effector activity of KIR3DS1+ NK cells is specifically targeted to HIV infected cells (via recognition of a HLA-Bw480I-dependent ligand), they may contribute to control virus replication, whereas if KIR3DS1+ cells are solely activated in a "bystander" fashion, they may predominantly mediate generalised immune activation, enhancing disease progression. Analysis of the *in vivo *importance of NK cell-mediated immunopathologic effects, the mechanisms by which they are mediated and the principal NK cell subset(s) involved is a priority for future work. This information will determine the feasibility of designing vaccine strategies to stimulate protective but not immunopathological NK responses, or to down-modulate immunopathological NK cell-mediated activity [115].

#### NKT cells

NKT cells are innate lymphocytes with properties of both NK cells and T cells, e.g. they express both a T cell receptor and markers characteristic of NK cells. Some NKT cells express relatively invariant TCRs that interact with ligands presented by the non-classical class I molecule CD1d, whilst others (typically defined as CD3+CD56+ lymphocytes in humans) express a much wider range of TCRs that interact with ligands presented by classical class I molecules.

Relatively little is known about NKT cell responses in acute and early HIV infection and the roles that these cells may be playing in protection and/or immunopathology. CD3+CD56+ NKT cells may contribute to control of HIV replication by mediating cytolysis of infected cells and/or via production of β-chemokines and other soluble factors; and both CD3+CD56+ and invariant NKT cells may mediate immunoregulatory effects that contribute to the initiation/enhancement of HIV-specific immune responses. Conversely, NKT cells may also mediate detrimental effects via promotion of immune activation and viral replication. Recent studies have shown that during acute SIV [116] and also acute HIV infection (Lavender, Borrow *et al*, unpublished) the frequency of circulating CD4+ NKT cells declines, likely as a consequence of virus infection, whilst CD8+ NKT cells increase in frequency, potentially as a result of antigen-driven expansion. Identification of the ligands recognized by NKT cells on HIV-infected cells is a priority for future studies, as is further analysis of the potential for employing these cells in an effector role in HIV vaccine design.

## Challenges faced in the development and evaluation of prophylactic or therapeutic strategies for achieving protection via modulation of innate responses

As discussed above, there is increasing understanding of the important role played by innate responses in both promoting and inhibiting HIV transmission, the establishment, dissemination and amplification of infection and the level at which viral replication is subsequently contained (Table 1). Although considerable work is still needed to define precisely which components of the innate response it would be desirable to up- or down-modulate to confer resistance to or enable better control of HIV replication, it is important that consideration also starts to be given to the even greater challenge of designing strategies via which modulation of selected innate responses could be achieved by vaccination or microbicides.

**Table 1:** Examples of protective and pathogenic effects mediated by innate responses at different stages of acute and early HIV-1 infection

Stage of infection	Beneficial effects	Detrimental effects
HIV transmission	SLPI, Elafin and defensins help to block HIV infection	SEVI and WFDC-1 enhance HIV infection

Establishment of initial foci of infection	Langerhans cells capture and destroy HIV virions Type 1 IFNs block HIV replication via upregulation of APOBECs and restriction factors	cDCs and macrophages act as sites for HIV replication and attract plus transmit infection to CD4+ T cells Local production of pro-inflammatory cytokines drives immune activation, enhancing viral replication

Local virus replication and spread to draining lymph nodes	Type 1 IFNs continue to limit HIV replication Locally-recruited innate effector cells combat virus replication	Proinflammatory cytokines, type 1 IFNs and WFDC-1 promote local immune activation, enhancing virus replication DC-SIGN+ DCs carry HIV to lymph nodes and infect CD4+ T cells

Further viral amplification and systemic dissemination	DCs activate innate effector cells including NK and NKT cells and begin to induce HIV-specific T cell responses NK, NKT and other innate effector cells combat HIV replication	cDCs and macrophages promote HIV transmission to CD4+ T cells DCs preferentially infect HIV-specific CD4+ T cells Immune activation mediated by pDCs and cDCs enhances HIV replication

Exponential virus growth and depletion of central memory CD4+ T cells	HIV replication is combated by type 1 IFNs, NK and NKT cells DCs promote induction of adaptive responses	Immune activation mediated by DCs, NK cells and NKT cells enhances HIV replication

Ongoing virus replication	Soluble innate factors and NK cells contribute to control of virus replication DCs help to sustain adaptive responses	cDCs and macrophages continue to promote HIV transmission to CD4+ T cells Immune activation mediated by DCs, NK cells and NKT cells enhances HIV replication

### a. Administration of innate effector molecules or factors that modulate the activity of innate subsets in microbicides or as therapeutic agents

The simplest method of employing innate defences for the control of HIV replication would be to directly administer innate antiviral effector molecules or the protective components thereof prophylactically in the form of microbicides, or therapeutically at a systemic level. Likewise protection could also be afforded by direct administration of agents able to block the activity of innate pathways that promote HIV replication. For example, recombinant forms of the active components of WAPs such as SLPI and Elafin, which are associated with reduced HIV transmission, could potentially be employed as microbicides or therapeutic agents; whilst blocking the activity of WFDC1/ps20, which promotes HIV spread, with monoclonal antibodies or peptide antagonists could represent an "anti-immunopathological" strategy.

An extension of this approach would be to directly administer substances known to modulate the effector activity of innate subsets. For example, HIV-specific monoclonal antibodies known to trigger antibody-dependent cell-mediated viral inhibition (ADCVI) responses by NK cells, macrophages or other effector cells could administered topically as part of a microbicide strategy, or be infused therapeutically in early HIV infection - an approach supported by the observation that the ability of one HIV-specific antibody, b12, to confer passive protection in a macaque vaginal simian/human immunodeficiency virus (SHIV) challenge model was dependent in part on its CD16-binding capacity [117]. Conversely anti-inflammatory agents could be included in microbicide preparations to reduce local immune activation, which is associated with HIV transmission.

### b. Use of vaccines to prime adaptive responses that will modulate subsequent innate effector functions

As the concept of using vaccines to stimulate the production of specific antibodies and memory T cell populations is well-established, the most straightforward approach to developing innate response-modulating vaccines may be to design vaccines to prime adaptive responses that will modulate innate effector functions in infected individuals. For example, vaccines could be developed to induce HIV-specific antibodies capable of mediating ADCVI. Notably, there was an inverse correlation between serum ADCVI antibody titres and HIV infection rate in the Vax 004 vaccine study [118]. Alternatively, vaccines could be used to elicit antibodies that block receptor-ligand interactions that have immunopathological consequences - e.g. interaction of the NKp44 ligand with NKp44 on NK cells.

Another possibility may be to develop vaccines or microbicides that can expand populations of regulatory T (Treg) cells that will dampen immune activation in HIV-infected individuals and reduce viral replication and spread. One study showed that CD4+CD25^high ^T cells that produced IL-10 in a HIVp24-specific fashion and could suppress the proliferation of HIVp24-specific CD4+CD25- T cells *in vitro *could be detected early after infection with HIV [119], suggesting that HIV-specific Treg activity can be induced (at least in the context of HIV infection). Furthermore, a negative correlation was observed between CD4+CD25^high ^T cell frequencies and the frequency of activated (HLA-DR+) CD4+ T cells in early infection [119], although whether this reflected a protective effect of the Treg cells or simply their preservation in the context of lower immune activation in acute/early infection was not determined. Nonetheless this remains an interesting area for future investigation.

### c. Use of vaccines to induce long-term alterations in innate subsets/functions

A more radical concept would be to use vaccines to modulate innate subsets/activity directly. One of the properties classically thought to distinguish innate and adaptive responses is that the former lack memory: but recent studies have challenged this view, providing evidence that NK cell responses can exhibit features characteristic of adaptive responses including "memory" [120-122]. One study demonstrated persistence of an elevated frequency of readily-triggered NK cells bearing a virus-specific receptor after infection in a murine cytomegalovirus model, and showed that the memory NK cells were capable of conferring protection on adoptive transfer [121]. Although evidence for similar "memory" NK responses in humans is currently lacking, and many questions still remain to be answered (such as how NK cell memory is generated and maintained and how long it lasts), these studies nonetheless raise the exciting prospect that it may be possible to expand populations of readily-triggered, HIV-reactive NK cells by vaccination. Another perhaps less hypothetical option may be to prime HIV-specific NKT cells to provide protection against infection. However, as discussed above, this would necessitate a better understanding of the activity mediated by NKT cells in HIV infection, and also of the ligands recognized by this innate subset on HIV-infected cells.

Another mechanism by which vaccines could induce long-lasting alterations in innate responses that would not require the existence of innate memory would be if the vaccine persisted and provided a source of continuous or repeated stimulation. There are a number of potential pitfalls to this approach, as chronic stimulation can have detrimental effects on immune functions; also maintenance of a generalized state of immune activation could enhance HIV acquisition/replication. However the possibility of providing an intermittent stimulus to retain selective innate responses at an elevated level remains worth exploring with caution.

### d. Evaluation of vaccination strategies

Given the potential (discussed in previous sections) for innate responses to mediate immunopathogenic effects in acute/early HIV infection, it is critically important that vaccination strategies designed to confer protection via modulation of innate responses are thoroughly tested to determine the full spectrum of innate functions altered and the potential for detrimental consequences. This is especially key where modulation of innate responses at mucosal sites is attempted, given the documented capacity of microbicide-induced modulation of local immune activation to enhance acquisition of infection (reviewed in [123]).

The effects of novel innate response-modulating vaccine strategies should ideally be tested in non-human primate immunodeficiency virus infection models prior to human administration. With this in mind, it is important that reagents appropriate for analyzing innate responses in non-human primates are developed, and the innate responses naturally activated in response to immunodeficiency virus infection characterized. Whilst work carried out to date in SIV-infected rhesus macaques indicates that there are many parallels between the innate responses activated here and in acute HIV-1 infection, there may also be some critical inter-species differences that may limit cross-species testing of certain vaccination strategies; this needs to be explored.

An as yet poorly-exploited avenue for gaining insight into the effects of vaccination strategies on innate responses and how these in turn may impact on protection is to analyze how vaccines that are currently in human trials may be modulating innate responses, how durable these effects are, and whether there is any impact on innate responses/functions during acute/early infection in vaccinated individuals who subsequently become infected. Given the difference in kinetics of activation of innate responses and the adaptive responses on which vaccine trials typically focus, this may require inclusion of extra post-vaccination sampling timepoints during vaccine trial design.

## Conclusions

Innate immunity plays important roles in mediating defence against HIV acquisition, control of infection following virus transmission and containment of virus replication during both the acute and chronic phases of infection. Conversely, innate responses can also have detrimental effects at all stages of infection, promoting virus transmission and the establishment of infection, enhancing subsequent virus replication and spread and contributing to CD4+ T cell destruction and disease progression. Importantly, recent studies have shown these opposing protective and pathogenic effects are mediated by every major component of the innate immune system (e.g. type 1 IFNs and other soluble factors, DCs and NK cells), prompting a need for more precise definition of the underlying molecular mechanisms so that they can be specifically up- or down-modulated by intervention strategies. Increasing evidence suggests that there may be potential to stimulate protective innate effector mechanisms to a level where HIV transmission and/or the establishment of disseminated infection is prevented. Likewise strategies for blocking generalised immune activation may also have prophylactic potential, and hold great promise as a novel therapeutic approach. The major challenge ahead lies in determining how these potentially important goals may be safely realized to yield novel infection and disease prevention strategies that will complement more traditional approaches. Some of the priorities for research in this area are summarised in Table 2.

**Table 2:** Strategies for targeting innate immunity to combat HIV infection and research priorities to advance their development

Strategy	Priorities for future research	Most rapidly-realised goals?
A. Development of microbicides and passive protection strategies that mediate defence at mucosal infection sites via deployment or local modulation of innate immunity	Structure-function studies to enable the design of small molecules that selectively induce the HIV-inhibitory properties of defensins, WAPs, etc	
		
	Identify the key mechanisms involved in type 1 IFN-mediated inhibition of HIV replication so that the pathways involved can be selectively invoked to block viral infection	
		
	Evaluate the effect of local administration of immunosuppressive agents at mucosal exposure sites on HIV acquisition	←

B. Design of vaccines to prime adaptive responses that mediate protection via modulation of innate effector functions	Clarify the importance of ADCVI activity as a means of antibody-mediated control of HIV infection; and Define the key characteristics of antibodies that induce strong ADCVI activity (e.g. isotype, glycosylation status, specificity, affinity)	←
		
	Verify the existence of HIV-specific Treg cells and determine their in vivo roles, particularly their impact on generalised immune activation	

C. Creation of strategies for achieving protection by directly inducing long-term alterations in innate subsets and/or their functions	Characterise NK cell memory in humans (e.g. NK populations involved, longevity, modes of induction); and Identify the ligands on HIV-infected cells that trigger NK cells mediating protective functions, to enable design of immunogens to stimulate these NK subsets	
		
	Analyse the roles of NKT cell subsets in protection versus pathogenesis during HIV infection, to determine the utility of targeting these cells in vaccine design	
		
	Explore the effects of persisting vaccine vectors on local and/or systemic innate responses	←

## List of abbreviations

ADCVI: antibody-dependent cell-mediated virus inhibition; AHI: acute HIV-1 infection; AID: activation-induced cytidine deaminase; AIDS: acquired immunodeficiency syndrome; APOBEC: apolipoprotein B editing complex, catalytic subunit (APOBEC); CCR: chemokine receptor; cDC: conventional DC (including myeloid DCs and other non-pDC types); DC: dendritic cell; DC-SIGN: dendritic cell-specific, intercellular adhesion molecule-grabbing non-integrin; GALT: gut-associated lymphoid tissue; HIV: human immunodeficiency virus; HLA: human leukocyte antigen; IDO: indoleamine 2,3-dioxygenase; IFN: interferon; IL: interleukin; IRF: interferon-regulatory factor; Ig: immunoglobulin; LARG: leukemia associated Rho guanine nucleotide-exchange factor; LPS: lipopolysaccharide; MHC: major histocompatibility complex; MIP: macrophage inflammatory protein; NF: nuclear factor; NK: natural killer; PBMC: peripheral blood mononuclear cell; pDC: plasmacytoid DC; PRR: pattern-recognition receptor; RIG-I: retinoic acid-inducible gene I; SEVI: semen-derived enhancer of virus infection; SIV: simian immunodeficiency virus; SHIV: simian/human immunodeficiency virus; SLPI: secretory leukocyte protease inhibitor; TLR: toll-like receptor; TNF: tumour necrosis factor; TRIM: tripartite motif; Treg: regulatory T; WAP: whey acidic protein; WFDC1: whey acidic protein four-disulfide core domain 1

## Competing interests

The authors declare that they have no competing interests.

## Authors' contributions

RJS, AV and PB organized the EUROPRISE workshop, where participants gave formal presentations and contributed to discussion. PB, RJS and AV drafted sections of the text, edited one another's contributions and read and approved the final manuscript.

## Authors' information

PB is a Reader in the Nuffield Dept of Clinical Medicine at the University of Oxford, UK whose research focuses on innate and T cell responses in persistent virus infections including HIV.

RJS is Professor of Cellular and Molecular Infection at St George's, University of London, UK. His work focuses on development of new prevention technology for HIV including vaccines and microbicides.

AV is a Senior Lecturer in the Department of Infectious Diseases at King's College London, UK whose research includes studies of virus/host interactions that regulate innate and adaptive T-cell immunity to HIV.

## References

[B1] Pitisuttithum P Gilbert P Gurwith M Heyward W Martin M van Griensven F Hu D Tappero JW Choopanya K Group. BVE Randomized, double-blind, placebo-controlled efficacy trial of a bivalent recombinant glycoprotein 120 HIV-1 vaccine among injection drug users in Bangkok, Thailand J Infect Dis 2006 194 1661 1671 10.1086/508748 17109337

[B2] Sekaly RP The failed HIV Merck vaccine study: a step back or a launching point for future vaccine development? J Exp Med 2008 205 7 12 10.1084/jem.20072681 18195078 PMC2234358

[B3] Rerks-Ngarm S Pitisuttithum P Nitayaphan S Kaewkungwal J Chiu J Paris R Premsri N Namwat C de Souza M Adams E Vaccination with ALVAC and AIDSVAX to prevent HIV-1 infection in Thailand N Engl J Med 2009 361 2209 2220 10.1056/NEJMoa0908492 19843557

[B4] Rhee EG Barouch DH Harnessing innate immunity for HIV vaccine development Clin Exp Immunol 2009 157 174 180 10.1111/j.1365-2249.2009.03928.x 19604256 PMC2730842

[B5] Harandi AM Medaglini D Shattock RJ EUROPRISE. WGcb Vaccine adjuvants: a priority for vaccine research Vaccine 2010 28 2363 2366 10.1016/j.vaccine.2009.12.084 20064476

[B6] Keele BF Giorgi EE Salazar-Gonzalez JF Decker JM Pham KT Salazar MG Sun C Grayson T Wang S Li H Identification and characterization of transmitted and early founder virus envelopes in primary HIV-1 infection Proc Natl Acad Sci USA 2008 105 7552 7557 10.1073/pnas.0802203105 18490657 PMC2387184

[B7] Münch J Rücker E Ständker L Adermann K Goffinet C Schindler M Wildum S Chinnadurai R Rajan D Specht A Semen-derived amyloid fibrils drastically enhance HIV infection Cell 2007 131 1059 1071 10.1016/j.cell.2007.10.014 18083097

[B8] Roan NR Münch J Arhel N Mothes W Neidleman J Kobayashi A Smith-McCune K Kirchhoff F Greene WC The cationic properties of SEVI underlie its ability to enhance human immunodeficiency virus infection J Virol 2009 83 73 80 10.1128/JVI.01366-08 18945786 PMC2612336

[B9] Kim KA Yolamanova M Zirafi O Roan NR Staendker L Forssmann WG Burgener A Dejucq-Rainsford N Hahn BH Shaw GM Semen-mediated enhancement of HIV infection is donor-dependent and correlates with the levels of SEVI Retrovirology 2010 7 55 10.1186/1742-4690-7-55 20573198 PMC2914040

[B10] Nakashima H Yamamoto N Masuda M Fuji iN Defensins inhibit HIV replication in vitro AIDS 1993 7 1129 10.1097/00002030-199308000-00019 8397954

[B11] Quiñones-Mateu ME Lederman MM Feng Z Chakraborty B Weber J Rangel HR Marotta ML Mirza M Jiang B Kiser P Human epithelial beta-defensins 2 and 3 inhibit HIV-1 replication AIDS 2003 17 F39 48 10.1097/00002030-200311070-00001 14571200

[B12] Guo CJ Tan N Song L Douglas SD Ho WZ Alpha-defensins inhibit HIV infection of macrophages through upregulation of CC-chemokines AIDS 2004 18 10.1097/00002030-200405210-00020 15166542 PMC4035112

[B13] Chang TL Vargas JJ DelPortillo A Klotman ME Dual role of alpha-defensin-1 in anti-HIV-1 innate immunity J Clin Invest 2005 115 765 773 15719067 10.1172/JCI200521948 PMC548697

[B14] Sun L Finnegan CM Kish-Catalone T Blumenthal R Garzino-Demo P La Terra Maggiore GM Berrone S Kleinman C Wu Z Abdelwahab S Human beta-defensins suppress human immunodeficiency virus infection: potential role in mucosal protection J Virol 2005 79 14318 14329 10.1128/JVI.79.22.14318-14329.2005 16254366 PMC1280242

[B15] Gallo SA Wang W Rawat SS Jung G Waring AJ Cole AM Lu H Yan X Daly NL Craik DJ Theta-defensins prevent HIV-1 Env-mediated fusion by binding gp41 and blocking 6-helix bundle formation J Biol Chem 2006 281 18787 18792 10.1074/jbc.M602422200 16648135

[B16] Furci L Sironi F Tolazzi M Vassena L Lusso P Alpha-defensins block the early steps of HIV-1 infection: interference with the binding of gp120 to CD4 Blood 2007 109 2928 2935 17132727 10.1182/blood-2006-05-024489

[B17] Klotman ME Rapista A Teleshova N Micsenyi A Jarvis GA Lu W Porter E Chang TL Neisseria gonorrhoeae-induced human defensins 5 and 6 increase HIV infectivity: role in enhanced transmission J Immunol 2008 180 6176 6185 18424739 10.4049/jimmunol.180.9.6176 PMC3042429

[B18] Territo MCGT Selsted ME Lehrer R Monocyte-chemotactic activity of defensins from human neutrophils J Clin Invest 1989 84 2017 2020 10.1172/JCI114394 2592571 PMC304087

[B19] Yang D Chertov O Bykovskaia SN Chen Q Buffo MJ Shogan J Anderson M Schröder JM Wang JM Howard OM Oppenheim JJ Beta-defensins: linking innate and adaptive immunity through dendritic and T cell CCR6 Science 1999 286 525 528 10.1126/science.286.5439.525 10521347

[B20] Yang D Chen Q Chertov O Oppenheim JJ Human neutrophil defensins selectively chemoattract naive T and immature dendritic cells J Leukoc Biol 2000 68 9 14 10914484

[B21] Funderburg N Lederman MM Feng Z Drage MG Jadlowsky J Harding CV Weinberg A Sieg SF Human -defensin-3 activates professional antigen-presenting cells via Toll-like receptors 1 and 2 Proc Natl Acad Sci USA 2007 104 18631 18635 10.1073/pnas.0702130104 18006661 PMC2141828

[B22] Shi J Aono S Lu W Ouellette AJ Hu X Ji Y Wang L Lenz S van Ginkel FW Liles M A novel role for defensins in intestinal homeostasis: regulation of IL-1beta secretion J Immunol 2007 179 237 243 10.4049/jimmunol.179.2.1245 17617617

[B23] Levinson P Kaul R Kimani J Ngugi E Moses S MacDonald KS Broliden K Hirbod T Group KHS Levels of innate immune factors in genital fluids: association of alpha defensins and LL-37 with genital infections and increased HIV acquisition AIDS 2009 23 309 317 10.1097/QAD.0b013e328321809c 19114868

[B24] Consortium CSaA Initial sequence of the chimpanzee genome and comparison with the human genome Nature 2005 437 69 87 10.1038/nature04072 16136131

[B25] Iqbal SM Ball TB Levinson P Maranan L Jaoko W Wachihi C Pak BJ Podust VN Broliden K Hirbod T Elevated elafin/trappin-2 in the female genital tract is associated with protection against HIV acquisition AIDS 2009 23 1669 1677 10.1097/QAD.0b013e32832ea643 19553806

[B26] Jana NK Gray LR Shugars DC Human immunodeficiency virus type 1 stimulates the expression and production of secretory leukocyte protease inhibitor (SLPI) in oral epithelial cells: a role for SLPI in innate mucosal immunity J Virol 2005 79 6432 6440 10.1128/JVI.79.10.6432-6440.2005 15858026 PMC1091668

[B27] Ma G Greenwell-Wild T Lei K Jin W Swisher J Hardegen N Wild CT Wahl SM Secretory leukocyte protease inhibitor binds to annexin II, a cofactor for macrophage HIV-1 infection J Exp Med 2004 200 1337 1346 10.1084/jem.20041115 15545357 PMC2211913

[B28] Py B Basmaciogullari S Bouchet J Zarka M Moura IC Benhamou M Monteiro RC Hocini H Madrid R Benichou S The phospholipid scramblases 1 and 4 are cellular receptors for the secretory leukocyte protease inhibitor and interact with CD4 at the plasma membrane PLoS ONE 2009 4 e5006 10.1371/journal.pone.0005006 19333378 PMC2659420

[B29] Alvarez R Reading J King DFL Hayes M Easterbrook P Ressler S Yang F Rowley D Vyakarnam A WFDC1/ps20 is a novel innate immunomodulatory signature protein of HIV permissive memory CD4 T-cells that promotes infection by up-regulating CD54 integrin expression and is elevated in HIV-1 infection J Virol 2008 82 471 486 10.1128/JVI.00939-07 17942534 PMC2224370

[B30] Bingle CD Vyakarnam A Novel innate immune functions of the whey acidic protein family Trends Immunol 2008 29 444 453 10.1016/j.it.2008.07.001 18676177

[B31] Xu W He B Chiu A Chadburn A Shan M Buldys M Ding A Knowles DM Santini PA Cerutti A Epithelial cells trigger frontline immunoglobulin class switching through a pathway regulated by the inhibitor SLPI Nat Immunol 2007 8 294 303 10.1038/ni1434 17259987

[B32] Conticello SG Langlois MA Yang Z Neuberger MS DNA deamination in immunity: AID in the context of its APOBEC relatives Adv Immunol 2007 94 37 73 full_text 17560271 10.1016/S0065-2776(06)94002-4

[B33] Jiang W Lederman MM Hunt P Sieg SF Haley K Rodriguez B Landay A Martin J Sinclair E Asher AI Plasma levels of bacterial DNA correlate with immune activation and the magnitude of immune restoration in persons with antiretroviral-treated HIV infection J Infect Dis 2009 199 1177 1185 10.1086/597476 19265479 PMC2728622

[B34] Ancuta P Kamat A Kunstman KJ Kim EY Autissier P Wurcel A Zaman T Stone D Mefford M Morgello S Microbial translocation is associated with increased monocyte activation and dementia in AIDS patients PLoS ONE 2008 3 e2516 10.1371/journal.pone.0002516 18575590 PMC2424175

[B35] Reddy S Vyakarnam A Conference on Retroviruses and Opportunistic Infections; San Francisco 2010

[B36] Yamamoto JK Barre-Sinoussi F Bolton V Pedersen NC Gardner MB Human alpha- and beta-interferon but not gamma- suppress the in vitro replication of LAV, HTLV-III and ARV-2 J Interferon Res 1986 6 143 152 2425014 10.1089/jir.1986.6.143

[B37] Pitha PM Multiple effects of interferon on the replication of human immunodeficiency virus type 1 Antiviral Res 1994 24 209 215 10.1016/0166-3542(94)90068-X 7526792

[B38] Tough DF Type I interferon as a link between innate and adaptive immunity through dendritic cell stimulation Leuk Lymphoma 2004 45 257 264 10.1080/1042819031000149368 15101709

[B39] Takeuchi H Matano T Host factors involved in resistance to retroviral infection Microbiol Immunol 2008 52 318 325 10.1111/j.1348-0421.2008.00040.x 18577167

[B40] Malim MH APOBEC proteins and intrinsic resistance to HIV-1 infection Philos Trans R Soc Lond B Biol Sci 2009 364 675 687 10.1098/rstb.2008.0185 19038776 PMC2660912

[B41] Towers GJ The control of viral infection by tripartite motif proteins and cyclophilin A Retrovirology 2007 4 40 10.1186/1742-4690-4-40 17565686 PMC1906832

[B42] Neil SJ Zang T Bieniasz PD Tetherin inhibits retrovirus release and is antagonized by HIV-1 Vpu Nature 2008 451 425 430 10.1038/nature06553 18200009

[B43] Malim MH Emerman M HIV-1 accessory proteins - ensuring viral survival in a hostile environment Cell Host Microbe 2008 3 388 398 10.1016/j.chom.2008.04.008 18541215

[B44] Neil SJ Bieniasz PD Human immunodeficiency virus, restriction factors, and interferon J Interferon Cytokine Res 2009 29 569 580 10.1089/jir.2009.0077 19694548 PMC2956573

[B45] Asaoka K Ikeda K Hishinuma T Horie-Inoue K Takeda S Inoue S A retrovirus restriction factor TRIM5alpha is transcriptionally regulated by interferons Biochem Biophys Res Commun 2005 338 1950 1956 10.1016/j.bbrc.2005.10.173 16289103

[B46] Chen K Huang J Zhang C Huang S Nunnari G Wang FX Tong X Gao L Nikisher K Zhang H Alpha interferon potently enhances the anti-human immunodeficiency virus type 1 activity of APOBEC3G in resting primary CD4 T cells J Virol 2006 80 7645 7657 10.1128/JVI.00206-06 16840343 PMC1563726

[B47] Peng G Le iKJ Jin W Greenwell-Wild T Wahl SM Induction of APOBEC3 family proteins, a defensive maneuver underlying interferon-induced anti-HIV-1 activity J Exp Med 2006 203 41 46 10.1084/jem.20051512 16418394 PMC2118075

[B48] Sakuma R Mael AA Ikeda Y Alpha interferon enhances TRIM5alpha-mediated antiviral activities in human and rhesus monkey cells J Virol 2007 81 10201 10206 10.1128/JVI.00419-07 17609277 PMC2045407

[B49] Neil SJ S V S WI Bieniasz PD An interferon alpha-induced tethering mechanism inhibits HIV-1 and Ebola virus particle release but is counteracted by the HIV-1 Vpu protein Cell Host Microbe 2007 2 193 203 10.1016/j.chom.2007.08.001 18005734 PMC3793644

[B50] Gendelman HE Baca LM Turpin J Kalter DC Hansen B Orenstein JM Dieffenbach CW Friedman RM Meltzer MS Regulation of HIV replication in infected monocytes by IFN-alpha. Mechanisms for viral restriction J Immunol 1990 145 2669 2676 1976701

[B51] Gendelman HE Friedman RM Joe S Baca LM Turpin JA Dveksler G Meltzer MS Dieffenbach C A selective defect of interferon alpha production in human immunodeficiency virus-infected monocytes J Exp Med 1990 172 1433 1442 10.1084/jem.172.5.1433 2264889 PMC2188659

[B52] Tsang J Chain BM Miller RF Webb BL Barclay W Towers GJ Katz DR Noursadeghi M HIV-1 infection of macrophages is dependent on evasion of innate immune cellular activation AIDS 2009 23 2255 2263 10.1097/QAD.0b013e328331a4ce 19741482 PMC2873676

[B53] Doehle BP Hladik F McNevin JP McElrath MJ Gale MJ Human immunodeficiency virus type 1 mediates global disruption of innate antiviral signaling and immune defenses within infected cells J Virol 2009 83 10395 10405 10.1128/JVI.00849-09 19706707 PMC2753137

[B54] Wang Y Bergmeier LA Stebbings R Seidl T Whittall T Singh M Berry N Almond N Lehner T Mucosal immunization in macaques upregulates the innate APOBEC 3G anti-viral factor in CD4+ memory T cells Vaccine 2009 27 870 881 10.1016/j.vaccine.2008.11.084 19084567 PMC2744409

[B55] Turelli P Liagre-Quazzola A Mangeat B Verp S Jost S Trono D APOBEC3-independent interferon-induced viral clearance in hepatitis B virus transgenic mice J Virol 2008 82 6585 6590 10.1128/JVI.00216-08 18434399 PMC2447049

[B56] Hirbod T Bailey RC Agot K Moses S Ndinya-Achola J Murugu R Andersson J Nilsson J Broliden K Abundant expression of HIV target cells and C-type lectin receptors in the foreskin tissue of young Kenyan men Am J Pathol 2010 176 2798 2805 10.2353/ajpath.2010.090926 20395432 PMC2877841

[B57] Hodges A Sharrocks K Edelmann M Baban D Moris A Schwartz O Drakesmith H Davies K Kessler B McMichael A Simmons A Activation of the lectin DC-SIGN induces an immature dendritic cell phenotype triggering Rho-GTPase activity required for HIV-1 replication Nat Immunol 2007 8 569 577 10.1038/ni1470 17496896

[B58] Gringhuis SI van der Vlist M van den Berg LM den Dunnen J Litjens M Geijtenbeek TB HIV-1 exploits innate signaling by TLR8 and DC-SIGN for productive infection of dendritic cells Nat Immunol 2010 11 419 426 10.1038/ni.1858 20364151

[B59] Gringhuis SI den Dunnen J Litjens M van der Vlist M Geijtenbeek TBH Carbohydrate-specific signaling through the DC-SIGN signalosome tailors immunity to Mycobacterium tuberculosis, HIV-1 and Helicobacter pylori Nat Immunol 2009 10 1081 1088 10.1038/ni.1778 19718030

[B60] de Witte L Nabatov A Pion M Fluitsma D de Jong MA de Gruijl T Piguet V van Kooyk Y Geijtenbeek TB Langerin is a natural barrier to HIV-1 transmission by Langerhans cells Nat Med 2007 13 367 371 10.1038/nm1541 17334373

[B61] Fahrbach KM Barry SM Ayehunie S Lamore S Klausner M Hope TJ Activated CD34-derived Langerhans cells mediate transinfection with human immunodeficiency virus J Virol 2007 81 6858 6868 10.1128/JVI.02472-06 17442711 PMC1933306

[B62] de Witte L Bobardt M Chatterji U Degeest G David G Geijtenbeek TB Gallay P Syndecan-3 is a dendritic cell-specific attachment receptor for HIV-1 Proc Natl Acad Sci USA 2007 104 19464 19469 10.1073/pnas.0703747104 18040049 PMC2148312

[B63] Li Q Estes JD Schlievert PM Duan L Brosnahan AJ Southern PJ Reilly CS Peterson ML Schultz-Darken N Brunner KG Glycerol monolaurate prevents mucosal SIV transmission Nature 2009 458 1034 1038 10.1038/nature07831 19262509 PMC2785041

[B64] Cameron PU Handley AJ Baylis DC Solomon AE Bernard N Purcell DF Lewin SR Preferential infection of dendritic cells during human immunodeficiency virus type 1 infection of blood leukocytes J Virol 2007 81 2297 2306 10.1128/JVI.01795-06 17166903 PMC1865918

[B65] Pion M Granelli-Piperno A Mangeat B Stalder R Correa R Steinman RM Piguet V APOBEC3G/3F mediates intrinsic resistance of monocyte-derived dendritic cells to HIV-1 infection J Exp Med 2006 203 2887 2893 10.1084/jem.20061519 17145955 PMC2118170

[B66] Loré K Smed-Sörensen A Vasudevan J Mascola JR Koup RA Myeloid and plasmacytoid dendritic cells transfer HIV-1 preferentially to antigen-specific CD4+ T cells J Exp Med 2005 201 2023 2033 10.1084/jem.20042413 15967828 PMC2212038

[B67] Groot F van Capel TM Kapsenberg ML Berkhout B de Jong EC Opposing roles of blood myeloid and plasmacytoid dendritic cells in HIV-1 infection of T cells: transmission facilitation versus replication inhibition Blood 2006 108 1957 1964 10.1182/blood-2006-03-010918 16705088

[B68] Beignon AS McKenna K Skoberne M Manches O DaSilva I Kavanagh DG Larsson M Gorelick RJ Lifson JD Bhardwaj N Endocytosis of HIV-1 activates plasmacytoid dendritic cells via Toll-like receptor-viral RNA interactions J Clin Invest 2005 115 3265 3275 10.1172/JCI26032 16224540 PMC1253628

[B69] Meier A Alter G Frahm N Sidhu H Li B Bagchi A Teigen N Streeck H Stellbrink HJ Hellman J MyD88-dependent immune activation mediated by human immunodeficiency virus type 1-encoded Toll-like receptor ligands J Virol 2007 81 8180 8191 10.1128/JVI.00421-07 17507480 PMC1951290

[B70] Fonteneau JF Larsson M Beignon AS McKenna K Dasilva I Amara A Liu YJ Lifson JD Littman DR Bhardwaj N Human immunodeficiency virus type 1 activates plasmacytoid dendritic cells and concomitantly induces the bystander maturation of myeloid dendritic cells J Virol 2004 78 5223 5232 10.1128/JVI.78.10.5223-5232.2004 15113904 PMC400371

[B71] Stacey AR Norris PJ Qin L Haygreen EA Taylor E Heitman J Lebedeva M DeCamp A Li D Grove D Induction of a striking systemic cytokine cascade prior to peak viremia in acute human immunodeficiency virus type 1 infection, in contrast to more modest and delayed responses in acute hepatitis B and C virus infections J Virol 2009 83 3719 3733 10.1128/JVI.01844-08 19176632 PMC2663284

[B72] Pacanowski J Kahi S Baillet M Lebon P Deveau C Goujard C Meyer L Oksenhendler E Sinet M Hosmalin A Reduced blood CD123+ (lymphoid) and CD11c+ (myeloid) dendritic cell numbers in primary HIV-1 infection Blood 2001 98 3016 3021 10.1182/blood.V98.10.3016 11698285

[B73] Malleret B Manéglier B Karlsson I Lebon P Nascimbeni M Perié L Brochard P Delache B Calvo J Andrieu T Primary infection with simian immunodeficiency virus: plasmacytoid dendritic cell homing to lymph nodes, type I interferon, and immune suppression Blood 2008 112 4598 4608 10.1182/blood-2008-06-162651 18787223

[B74] Brown KN Wijewardana V Liu X Barratt-Boyes SM Rapid influx and death of plasmacytoid dendritic cells in lymph nodes mediate depletion in acute simian immunodeficiency virus infection PLoS Pathog 2009 5 e1000413 10.1371/journal.ppat.1000413 19424421 PMC2671605

[B75] Loré K Sönnerborg A Broström C Goh LE Perrin L McDade H Stellbrink HJ Gazzard B Weber R Napolitano LA Accumulation of DC-SIGN+CD40+ dendritic cells with reduced CD80 and CD86 expression in lymphoid tissue during acute HIV-1 infection AIDS 2002 16 683 692 10.1097/00002030-200203290-00003 11964524

[B76] Shiina M Rehermann B Cell culture-produced hepatitis C virus impairs plasmacytoid dendritic cell function Hepatology 2008 47 385 395 10.1002/hep.21996 18064579

[B77] Liang H Russell RS Yonkers NL McDonald D Rodriguez B Harding CV Anthony DD Differential effects of hepatitis C virus JFH1 on human myeloid and plasmacytoid dendritic cells J Virol 2009 83 5693 5707 10.1128/JVI.02671-08 19297478 PMC2681964

[B78] Herbeuval JP Hardy AW Boasso A Anderson SA Dolan MJ Dy M Shearer GM Regulation of TNF-related apoptosis-inducing ligand on primary CD4+ T cells by HIV-1: role of type 1 IFN-producing plasmacytoid dendritic cells Proc Natl Acad Sci USA 2005 102 13974 13979 10.1073/pnas.0505251102 16174727 PMC1224361

[B79] Mueller YM Do DH Altork SR Artlett CM Gracely EJ Katsetos CD Legido A Villinger F Altman JD Brown CR IL-15 treatment during acute simian immunodeficiency virus (SIV) infection increases viral set point and accelerates disease progression despite the induction of stronger SIV-specific CD8+ T cell responses J Immunol 2008 180 350 360 18097036 10.4049/jimmunol.180.1.350 PMC2929904

[B80] Meier A Chang JJ Chan ES Pollard RB Sidhu HK Kulkarni S Wen TF Lindsay RJ Orellana L Mildvan D Sex differences in the Toll-like receptor-mediated response of plasmacytoid dendritic cells to HIV-1 Nat Med 2009 15 955 959 10.1038/nm.2004 19597505 PMC2821111

[B81] Estes JD Gordon SN Zeng M Chahroudi AM Dunham RM Staprans SI Reilly CS Silvestri G Haase AT Early resolution of acute immune activation and induction of PD-1 in SIV-infected sooty mangabeys distinguishes nonpathogenic from pathogenic infection in rhesus macaques J Immunol 2008 180 6798 6807 18453600 10.4049/jimmunol.180.10.6798 PMC2596686

[B82] Diop OM Ploquin MJ Mortara L Faye A Jacquelin B Kunkel D Lebon P Butor C Hosmalin A Barré-Sinoussi F Müller-Trutwin MC Plasmacytoid dendritic cell dynamics and alpha interferon production during Simian immunodeficiency virus infection with a nonpathogenic outcome J Virol 2008 82 5145 5152 10.1128/JVI.02433-07 18385227 PMC2395206

[B83] Bosinger SE Li Q Gordon SN Klatt NR Duan L Xu L Francella N Sidahmed A Smith AJ Cramer EM Global genomic analysis reveals rapid control of a robust innate response in SIV-infected sooty mangabeys J Clin Invest 2009 119 3556 3572 19959874 10.1172/JCI40115 PMC2786806

[B84] Jacquelin B Mayau V Targat B Liovat AS Kunkel D Petitjean G Dillies MA Roques P Butor C Silvestri G Nonpathogenic SIV infection of African green monkeys induces a strong but rapidly controlled type I IFN response J Clin Invest 2009 119 3544 3555 19959873 10.1172/JCI40093 PMC2786805

[B85] Lederer S Favre D Walters KA Proll S Kanwar B Kasakow Z Baskin CR Palermo R McCune JM Katze MG Transcriptional profiling in pathogenic and non-pathogenic SIV infections reveals significant distinctions in kinetics and tissue compartmentalization PLoS Pathog 2009 5 e1000296 10.1371/journal.ppat.1000296 19214219 PMC2633618

[B86] Fahey JL Taylor JM Detels R Hofmann B Melmed R Nishanian P Giorgi JV The prognostic value of cellular and serologic markers in infection with human immunodeficiency virus type 1 N Engl J Med 1990 322 166 172 10.1056/NEJM199001183220305 1967191

[B87] Hazenberg MD Otto SA van Benthem BH Roos MT Coutinho RA Lange JM Hamann D Prins M Miedema F Persistent immune activation in HIV-1 infection is associated with progression to AIDS AIDS 2003 17 1881 1888 10.1097/00002030-200309050-00006 12960820

[B88] Durudas A Milush JM Chen HL Engram JC Silvestri G Sodora DL Elevated levels of innate immune modulators in lymph nodes and blood are associated with more-rapid disease progression in simian immunodeficiency virus-infected monkeys J Virol 2009 83 12229 12240 10.1128/JVI.01311-09 19759147 PMC2786739

[B89] Baenziger S Heikenwalder M Johansen P Schlaepfer E Hofer U Miller RC Diemand S Honda K Kundig TM Aguzzi A Speck RF Triggering TLR7 in mice induces immune activation and lymphoid system disruption, resembling HIV-mediated pathology Blood 2009 113 377 388 10.1182/blood-2008-04-151712 18824599

[B90] Mandl JN Barry AP Vanderford TH Kozyr N Chavan R Mucking S Barrat FJ Coffman RL Staprans SI Feinberg MB Divergent TLR7 and TLR9 signaling and type I interferon production distinguish pathogenic and nonpathogenic AIDS virus infections Nat Med 2008 14 1077 1087 10.1038/nm.1871 18806803

[B91] Campillo-Gimenez L Laforge M Fay M Brussel A Cumont MC Monceaux V Diop O Lévy Y Hurtrel B Zaunders J Nonpathogenesis of simian immunodeficiency virus infection is associated with reduced inflammation and recruitment of plasmacytoid dendritic cells to lymph nodes, not to lack of an interferon type I response, during the acute phase J Virol 2010 84 1838 1846 10.1128/JVI.01496-09 19939930 PMC2812402

[B92] Kornfeld C Ploquin MJ Pandrea I Faye A Onanga R Apetrei C Poaty-Mavoungou V Rouquet P Estaquier J Mortara L Antiinflammatory profiles during primary SIV infection in African green monkeys are associated with protection against AIDS J Clin Invest 2005 115 1082 1091 15761496 10.1172/JCI23006 PMC1062895

[B93] Kirchoff F Is the high virulence of HIV-1 an unfortunate coincidence of primate lentiviral evolution? Nat Rev Microbiol 2009 7 467 476 19305418 10.1038/nrmicro2111

[B94] Granelli-Piperno A Golebiowska A Trumpfheller C Siegal FP Steinman RM HIV-1-infected monocyte-derived dendritic cells do not undergo maturation but can elicit IL-10 production and T cell regulation Proc Natl Acad Sci USA 2004 101 7669 7674 10.1073/pnas.0402431101 15128934 PMC419664

[B95] Boasso A Herbeuval JP Hardy AW Anderson SA Dolan MJ Fuchs D Shearer GM HIV inhibits CD4+ T-cell proliferation by inducing indoleamine 2,3-dioxygenase in plasmacytoid dendritic cells Blood 2007 109 3351 3359 10.1182/blood-2006-07-034785 17158233 PMC1852248

[B96] Manches O Munn D Fallahi A Lifson J Chaperot L Plumas J Bhardwaj N HIV-activated human plasmacytoid DCs induce Tregs through an indoleamine 2,3-dioxygenase-dependent mechanism J Clin Invest 2008 118 3431 3439 10.1172/JCI34823 18776940 PMC2528911

[B97] Alter G Teigen N Ahern R Streeck H Meier A Rosenberg ES Altfeld M Evolution of innate and adaptive effector cell functions during acute HIV-1 infection J Infect Dis 2007 195 1452 1460 10.1086/513878 17436225

[B98] Alter G Rihn S Walter K Nolting A Martin M Rosenberg ES Miller JS Carrington M Altfeld M HLA class I subtype-dependent expansion of KIR3DS1+ and KIR3DL1+ NK cells during acute human immunodeficiency virus type 1 infection J Virol 2009 83 6798 6805 10.1128/JVI.00256-09 19386717 PMC2698561

[B99] Cohen GB Gandhi RT Davis DM Mandelboim O Chen BK Strominger JL Baltimore D The selective downregulation of class I major histocompatibility complex proteins by HIV-1 protects HIV-infected cells from NK cells Immunity 1999 10 661 671 10.1016/S1074-7613(00)80065-5 10403641

[B100] Bonaparte MI Barker E Killing of human immunodeficiency virus-infected primary T-cell blasts by autologous natural killer cells is dependent on the ability of the virus to alter the expression of major histocompatibility class I molecules Blood 2004 104 2087 2094 10.1182/blood-2004-02-0696 15117765

[B101] Alter G Martin MP Teigen N Carr WH Suscovich TJ Schneidewind A Streeck H Waring M Meier A Brander C Differential natural killer cell-mediated inhibition of HIV-1 replication based on distinct KIR/HLA subtypes J Exp Med 2007 204 3027 3036 10.1084/jem.20070695 18025129 PMC2118524

[B102] Martin MP Gao X Lee JH Nelson GW Detels R Goedert JJ Buchbinder S Hoots K Vlahov D Trowsdale J Epistatic interaction between *KIR3DS1 *and *HLA-B *delays the progression to AIDS Nat Genet 2002 31 429 434 12134147 10.1038/ng934

[B103] Qi Y Martin MP Gao X Jacobson L Goedert JJ Buchbinder S Kirk GD O'Brien SJ Trowsdale J Carrington M *KIR/HLA *pleiotropism: protection against both HIV and opportunistic infections PLoS Pathogens 2006 2 741 745 10.1371/journal.ppat.0020079 PMC1550271 16933987

[B104] Martin MP Qi Y Gao X Yamada E Martin JN Pereyra F Colombo S Brown EE Shupert WL Phair J Innate partnership of *HLA-B *and *KIR3DL1 *subtypes against HIV-1 Nat Genet 2007 39 733 740 10.1038/ng2035 17496894 PMC4135476

[B105] Bostik P Kobkitjaroen J Tang W Villinger F Pereira L Little DM Stephenson ST Bouzyk M Ansari AA Decreased NK cell frequency and function is associated with increased risk of KIR3DL allele polymorphism in simian immunodeficiency virus-infected rhesus macaques with high viral loads J Immunol 2009 182 3638 3649 10.4049/jimmunol.0803580 19265142 PMC5407377

[B106] Scott-Algara D Truong LX Versmisse P David A Luong TT Nguyen NV Theodorou I Barre-Sinoussi F Pancino G Cutting edge: increased NK cell activity in HIV-1-exposed but uninfected Vietnamese intravascular drug users J Immunol 2003 171 5663 5667 14634071 10.4049/jimmunol.171.11.5663

[B107] Montoya CJ Velilla PA Chougnet C Landay AL Rugeles MT Increased IFN-gamma production by NK and CD3+/CD56+ cells in sexually HIV-1-exposed but uninfected individuals Clin Immunol 2006 120 138 146 10.1016/j.clim.2006.02.008 16624619

[B108] Ravet S Scott-Algara D Bonnet E Tran HK Tran T Nguyen N Truong LX Theodorou I Barre-Sinoussi F Pancino G Paul P Distinctive NK-cell receptor repertoires sustain high-level constitutive NK-cell activation in HIV-exposed uninfected individuals Blood 2007 109 4296 4305 10.1182/blood-2006-08-040238 17272507

[B109] Boulet S Sharafi S Simic N Bruneau J Routy JP Tsoukas CM Bernard NF Increased proportion of KIR3DS1 homozygotes in HIV-exposed uninfected individuals AIDS 2008 22 595 599 10.1097/QAD.0b013e3282f56b23 18317000

[B110] Boulet S Song R Kamya P Bruneau J Shoukry NH Tsoukas CM Bernard NF HIV protective *KIR3DL1 *and *HLA-B *genotypes influence NK cell function following stimulation with HLA-devoid cells J Immunol 2010 184 2057 2064 10.4049/jimmunol.0902621 20061407

[B111] Colonna M Interleukin-22-producing natural killer cells and lymphoid tissue inducer-like cells in mucosal immunity Immunity 2009 31 15 23 10.1016/j.immuni.2009.06.008 19604490

[B112] Fausther-Bovendo H Sol-Foulon N Candotti D Agut H Schwartz O Debre P Vielliard V HIV escape from natural killer cell cytotoxicity: nef inhibits NKp44L expression on CD4+ T cells AIDS 2009 23 1077 1087 10.1097/QAD.0b013e32832cb26b 19424050

[B113] Gaudieri S DeSantis D McKinnon E Moore C Nolan D Witt CS Mallal SA Christiansen FT Killer immunoglobulin-like receptors and HLA act both independently and synergistically to modify HIV disease progression Genes Immun 2005 6 683 690 16121209 10.1038/sj.gene.6364256

[B114] Long BR Ndhlovu LC Oksenberg JR Lanier LL Hecht FM Nixon DF Barbour JD Conferral of enhanced natural killer cell function by KIR3DS1 in early human immunodeficiency virus type 1 infection J Virol 2008 82 4785 4792 10.1128/JVI.02449-07 18305035 PMC2346752

[B115] Vieillard V Le Grand R Dausset J Debre P A vaccine strategy against AIDS: an HIV gp41 peptide immunization prevents NKp44L expression and CD4+ T cell depletion in SHIV-infected macaques Proc Natl Acad Sci USA 2008 105 2100 2104 10.1073/pnas.0711629105 18234855 PMC2542869

[B116] Fernandez CS Chan AC Kyparissoudis K De Rose R Godfrey DI Kent SJ Peripheral NKT cells in simian immunodeficiency virus-infected macaques J Virol 2009 83 1617 1624 10.1128/JVI.02138-08 19052081 PMC2643790

[B117] Hessell AJ Hangartner L Hunter M Havenith CE Beurskens FJ Bakker JM Lanigan CM Landucci G Forthal DN Parren PW Fc receptor but not complement binding is important in antibody protection against HIV Nature 2007 449 101 104 10.1038/nature06106 17805298

[B118] Forthal DN Gilbert PB Landucci G Phan T Recombinant gp120 vaccine-induced antibodies inhibit clinical strains of HIV-1 in the presence of Fc receptor-bearing effector cells and correlate inversely with HIV infection rate J Immunol 2007 178 6596 6603 17475891 10.4049/jimmunol.178.10.6596

[B119] Kared H Lelièvre JD Donkova-Petrini V Aouba A Melica G Balbo M Weiss L Lévy Y HIV-specific regulatory T cells are associated with higher CD4 cell counts in primary infection AIDS 2008 22 2451 2460 10.1097/QAD.0b013e328319edc0 19005268 PMC3195674

[B120] O'Leary JG Goodarzi M Drayton DL von Andrian UH T cell- and B cell-independent adaptive immunity mediated by natural killer cells Nat Immunol 2006 7 507 516 10.1038/ni1332 16617337

[B121] Sun JC Beilke JN Lanier LL Adaptive immune features of natural killer cells Nature 2009 457 557 561 10.1038/nature07665 19136945 PMC2674434

[B122] Cooper MA Elliott JM Keyel PA Yang L Carrero JA Yokoyama WM Cytokine-induced memory-like natural killer cells Proc Natl Acad Sci USA 2009 106 1915 1919 10.1073/pnas.0813192106 19181844 PMC2644138

[B123] Cummins JEJ Doncel GF Biomarkers of cervicovaginal inflammation for the assessment of microbicide safety Sex Transm Dis 2009 36 S84 91 10.1097/OLQ.0b013e3181994191 19218890

